# High-Density 1R/1W Dual-Port Spin-Transfer Torque MRAM

**DOI:** 10.3390/mi13122224

**Published:** 2022-12-15

**Authors:** Yeongkyo Seo, Kon-Woo Kwon

**Affiliations:** 1 Department of Information and Communication Engineering, Inha University, Incheon 22212, Republic of Korea; 2 Department of Computer Engineering, Hongik University, Seoul 04066, Republic of Korea

**Keywords:** STT-MRAM, dual port, 1-Read/1-Write, area optimization, bit interleaving, simultaneous access conflict

## Abstract

Spin-transfer torque magnetic random-access memory (STT-MRAM) has several desirable features, such as non-volatility, high integration density, and near-zero leakage power. However, it is challenging to adopt STT-MRAM in a wide range of memory applications owing to the long write latency and a tradeoff between read stability and write ability. To mitigate these issues, an STT-MRAM bit cell can be designed with two transistors to support multiple ports, as well as the independent optimization of read stability and write ability. The multi-port STT-MRAM, however, is achieved at the expense of a higher area requirement due to an additional transistor per cell. In this work, we propose an area-efficient design of 1R/1W dual-port STT-MRAM that shares a bitline between two adjacent bit cells. We identify that the bitline sharing may cause simultaneous access conflicts, which can be effectively alleviated by using the bit-interleaving architecture with a long interleaving distance and the sufficient number of word lines per memory bank. We report various metrics of the proposed design based on the bit cell design using a 45 nm process. Compared to a standard single-port STT-MRAM, the proposed design shows a 15% lower read power and a 19% higher read-disturb margin. Compared with prior work on the 1R/1W dual-port STT-MRAM, the proposed design improves the area by 25%.

## 1. Introduction

**Figure 1:**
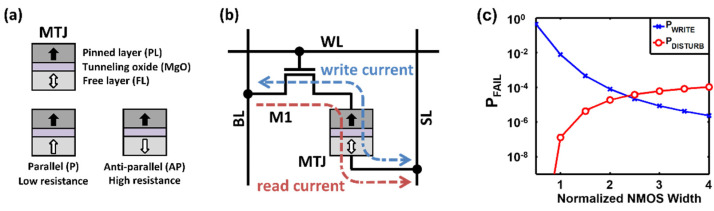
(**a**) Device structure for MTJ, (**b**) STT-MRAM bit cell structure, (**c**) probability of read-disturb failure and write failure, with respect to the normalized width of the access transistor [13].

Spin-transfer torque magnetic random-access memory (STT-MRAM) has drawn great attention as a promising candidate for future on-chip memory because of its desirable features such as high integration density, near-zero leakage power, non-volatility, and compatibility with the CMOS fabrication process [1,2,3,4,5,6,7,8,9]. A standard STT-MRAM bit cell comprises a single access transistor and a magnetic tunnel junction (MTJ) that functions as a storage element. The MTJ consists of a pinned layer (PL) and a free layer (FL) sandwiching a tunneling oxide barrier, as shown in Figure 1a. The magnetization of the PL is pinned to one direction, whereas the FL’s magnetization can be altered by passing an electrical current so that its direction is either parallel (P) or anti-parallel (AP) to that of the PL [1]. Since an MTJ resistance in the AP state is higher than that in the P state, a read operation can be performed by sensing the resistance of the MTJ. STT-MRAM is capable of >2x integration density, in comparison with conventional static RAM (SRAM) that requires six transistors per cell. Moreover, STT-MRAM can lower the total power dissipation by eliminating the leakage power because the MTJ is non-volatile [10].

Despite the aforementioned advantages, two major issues need to be addressed in order to adopt STT-MRAM in a wide range of memory applications. First, STT-MRAM has a high write latency that may degrade system performance [11,12]. When a read request occurs during a write operation, it may be delayed until the long-latency write is completed [12]. Second, the read current path is identical to the write current path, as shown in Figure 1b, creating a tradeoff between read stability and write ability [11]. For instance, if the access transistor has a large width for a reliable write operation, it is likely that an inadvertent bit flip occurs during a read operation, as depicted in Figure 1c [4].

**Figure 2:**
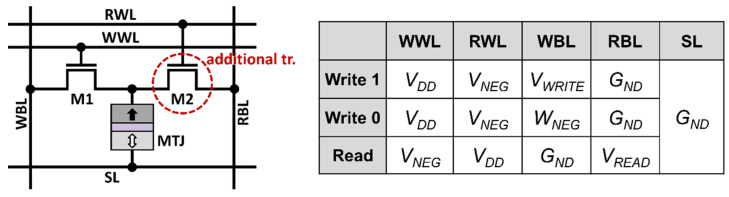
Dual 1R/1W STT-MRAM bit cell and biasing condition for write and read operations.

A possible solution is to design an STT-MRAM bit cell with multiple ports, such as one read and one write (1R/1W) dual-port STT-MRAM [11]. As shown in Figure 2, read and write operations can be simultaneously performed by using two different access transistors, and thus, the impact of a slow write operation can be effectively mitigated. Because a read-access transistor and a write-access transistor are separated, the read stability and write ability can be independently optimized. However, due to the requirement of an additional transistor, the multi-port design degrades the achievable memory density. Thus, the 1R/1W dual-port STT-MRAM trades off the write latency and the memory cell area.

In this paper, we propose an area-efficient design for 1R/1W dual-port STT-MRAM to improve the integration density. The proposed dual-port STT-MRAM shares a bitline between two adjacent bit cells. We identify that the bitline sharing may cause erroneous operations due to a creation of sneak path currents or a conflicting requirement of biasing conditions. We categorize such erroneous operations into three cases and show that each case can be mitigated by using the bit-interleaving architecture with long interleaving distance and the sufficient number of word lines per memory bank. Our simulation results show that the proposed designs can reduce the memory cell area by 25% in comparison with the prior 1R/1W STT-MRAM design shown in [11], at the same specification of 10 ns switching time, 20% write margin, and >35% read margin. Moreover, the proposed design achieves a 15% lower read power and a 19% higher read-disturb margin compared to the standard single-port STT-MRAM.

## 2. Review of Conventional STT-MRAMs

### 2.1. Single-Port STT-MRAM

**Figure 3:**
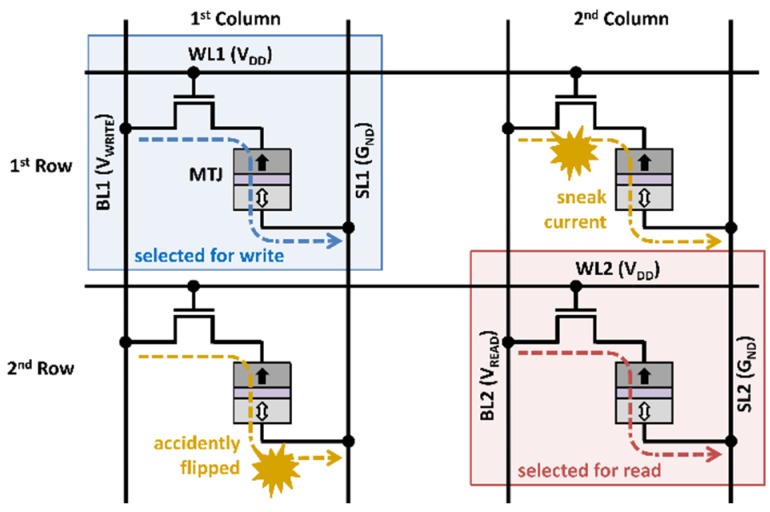
Simultaneous access conflict in single-port STT-MRAM in distance-2 bit-interleaving architecture.

In a conventional STT-MRAM with a single-port, when a write operation is being performed, read requests are delayed until the write operation is completed. This may result in performance degradation, especially for write-intensive applications [6]. Consider a 2 × 2 array of single-port STT-MRAM with a distance-2 bit-interleaving architecture, as shown in Figure 3. Each row is composed of bit cells connected with the same word line (WL), while each column is composed of bit cells connected with the same set of bitline (BL) and source line (SL). Moreover, an odd-column cell and an even-column cell cannot be contained in the same word due to column selection by distance-2 bit-interleaving [14,15,16,17]. Accordingly, all four bit cells shown in Figure 3 belong to different words. Now, it is easy to observe that simultaneous write and read operations are not allowed. For instance, to write a value 1 to a bit cell on the first column of first row, BL1 is set to the write voltage level (*V_WRITE_*), SL1 is grounded, and WL1 is asserted high. If the bit cell on the second column of the second row is accessed for a read operation at the same time, BL2 is biased at *V_READ_*, SL2 at *G_ND_*, and WL2 is asserted high. Note that the unselected cell in the first column of the second row may accidently flip its MTJ value because the activation of WL2 passes an electrical current from BL1 to SL1. It should be also noted that the read operation in the second column of the second row may be erroneous because the unwanted current flows through the unselected cell in the second column of the first row.

### 2.2. 1R/1W Dual-Port STT-MRAM

**Figure 4:**
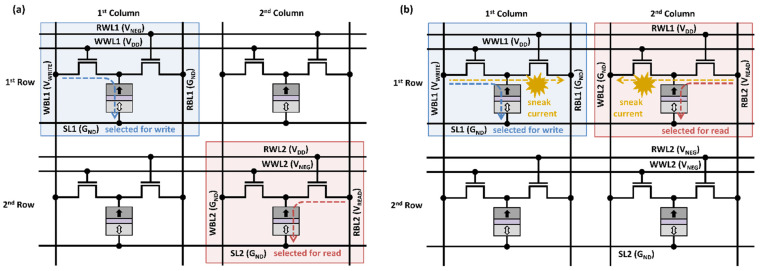
(**a**) Simultaneous write and read accesses in a 2 × 2 array of 1R/1W STT-MRAM in distance-2 bit-interleaving architecture; (**b**) a simultaneous access conflict occurred when accessing different words in a same row.

To avoid the aforementioned conflicts regarding simultaneous memory accesses, 1R/1W dual-port STT-MRAM was proposed in [11], in which an extra access transistor is required. For a write operation, the transistor M1 is activated by biasing write bitline (WBL) and write word line (WWL), as shown in Figure 2. For a read operation, the other transistor M2 is activated by biasing read bitline (RBL) and read word line (RWL), appropriately. The dedicated dual ports enable simultaneous write and read operations when two memory accesses are in different rows. To see this, consider a 2 × 2 array of 1R/1W STT-MRAM with a distance-2 bit-interleaving architecture, as shown in Figure 4. To write a bit cell on the first column of the first row, WBL1 is set to *V_WRITE_*, and WWL1 is asserted high. If the bit cell on the second column of the second row is accessed for a read operation at the same time, RBL2 is biased at *V_READ_*, and RWL2 is asserted high. In such a case, RWL1 and WWL2 remain de-asserted, which can prevent a current flow in the unselected bit cells.

However, we identify that simultaneous write and read operations are not supported when two memory accesses are attempted on different words in a same row [7,14]. See Figure 4b, where two adjacent bit-cells (that belong to different words) on the first row are being accessed, one cell for a write and the other cell for a read. Then, both RWL and WWL on the same row must be asserted high, creating sneak path currents. We define such a case as a *simultaneous access conflict*.

## 3. Proposed Design

**Figure 5:**
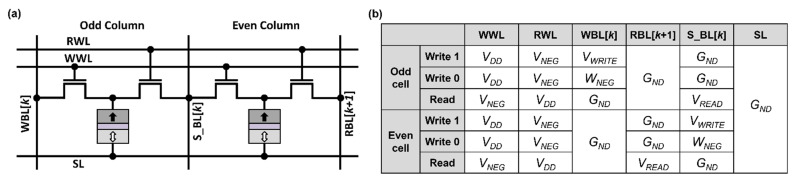
(**a**) Proposed 1R/1W STT-MRAM bit-cell schematic and (**b**) biasing condition for write and read operations.

In order to improve bit-cell area while supporting 1R/1W accesses, we propose a new design that shares a vertical BL between two adjacent bit cells. The shared BL, termed S_BL, is used as the RBL for an odd-column bit cell and as the WBL for an even-column bit cell, as shown in Figure 5a. Detailed biasing conditions for writing and reading the proposed bit cell are presented in Figure 5b, where SL is fixed at *G_ND_*, as is in the case of the prior design of 1R/1W STT-MRAM [11]. Note that for writing a value of zero, the WBL is biased at the negative voltage (*V_NEG_*), such that the current flows from the SL to the WBL via the MTJ. The WLs for unselected cells are also biased at *V_NEG_* to keep the access transistors in the unselected cells turned off [10].

Due to sharing of S_BL between two bit cells, as a trade-off, the proposed 1R/1W STT-MRAM may cause simultaneous access conflicts with higher probability. Simultaneous access conflicts due to the proposed design can be categorized into three cases:

(*Case 1*) Accesses to a *same row*.

**Figure 6:**
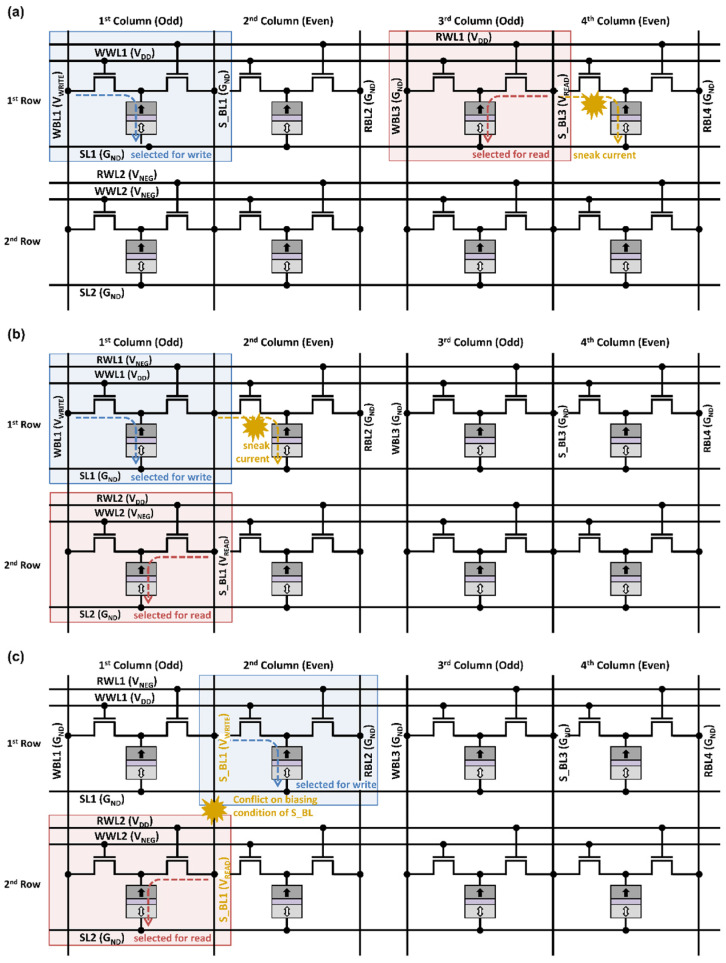
Simultaneous access conflicts by the proposed design: (**a**) *Case 1* by simultaneous accesses to a same row, (**b**) *Case 2* by simultaneous accesses to a same column, and (**c**) *Case 3* by simultaneous accesses to odd-column cell for a write and even-column cell for a read that share S_BL.

An example of *Case 1* is illustrated in Figure 6a, where both WWL1 and RWL1 are asserted high to write the cell in the first column of the first row and simultaneously read the cell in the third column of the first row. This can cause an unintended path for current flow on unselected cells, for example, the cells in the fourth column in the first row. The simultaneous access conflicts occurring in the prior design in [11] correspond to those in *Case 1*.

(*Case 2*) Accesses to a *same column*.

An example of *Case 2* is illustrated in Figure 6b, where WWL1 is asserted high, and S_BL1 is at V_READ_ to write the cell in the first column of the first row and simultaneously read the cell in the first column of the second row. This can cause an unintended current flow in the unselected cell in the second column of the first row.

(*Case 3*) Accesses to odd-column cell for a write and even-column cell for a read that share S_BL.

An example of *Case 3* is illustrated in Figure 6c, where S_BL1 should be at *V_WRITE_* to write an odd-column cell. At the same time, S_BL1 should be at *V_READ_* for reading an even-column cell, which is a conflict.

**Figure 7:**
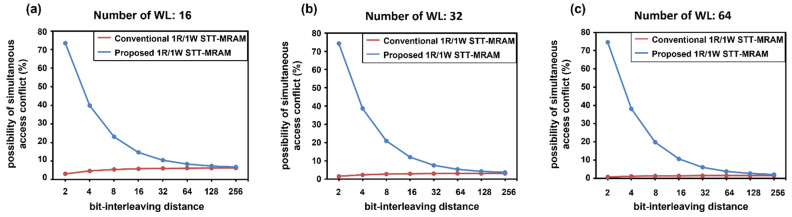
Probability of simultaneous access conflict with respect to the bit-interleaving distance when the number of WLs per bank is (**a**) 16, (**b**) 32, and (**c**) 64, respectively.

Figure 7 shows the possibility of simultaneous access conflicts with the assumption of uniformly random memory accesses. First, it is observed that the proposed design incurs simultaneous access conflicts with higher probability when compared to the conventional design in [11] that incurs simultaneous access conflicts only in *Case 1*. Specifically, if the distance-2 bit-interleaving is applied, occurrences of *Case 2* or *Case 3* are dominant over *Case 1*, leading to the large gap between the conventional design and the proposed design in terms of the probability of simultaneous access conflicts. However, increasing the interleaving distance can come to the rescue to reduce the probability of *Case 2* or *Case 3*; it can be observed that the probability of the conflict caused by *Case 2* or *Case 3* can be lowered by nearly half as the interleaving distance increases by a factor of two. Second, doubling the number of WLs per bank reduces the probability of *Case 1* by half, lowering the probability of simultaneous access conflicts for both the conventional design and the proposed design. Hence, simultaneous access conflicts of the proposed design can be effectively mitigated by using the long interleaving distance and the sufficient number of WLs per bank.

## 4. Layout Analysis

**Figure 8:**
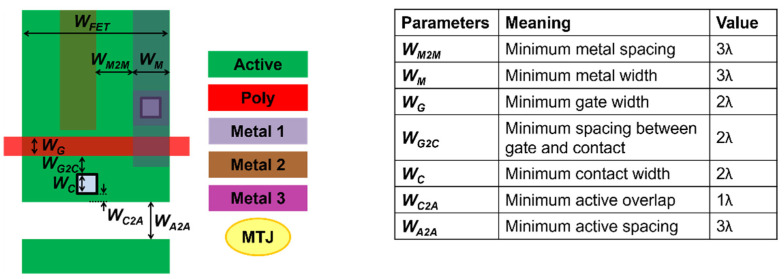
Parameters for the layout design rules.

**Figure 9:**
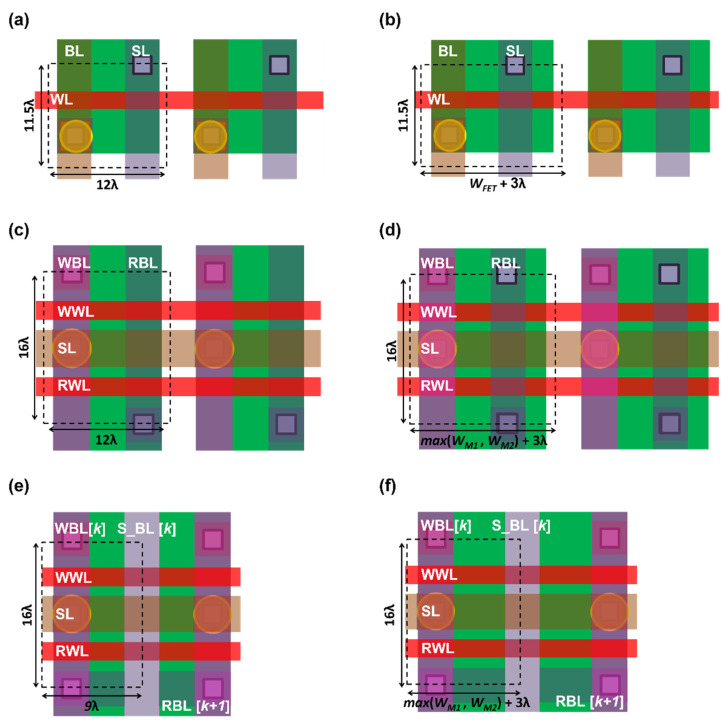
Single-port STT-MRAM layout (**a**) when the access transistor width is smaller than 9λ, (**b**) when its access transistor width is greater than 9λ, conventional 1R/1W STT-MRAM layout (**c**) when width of both transistors is smaller than 9λ, (**d**) when width of either of M1 and M2 is greater than 9λ, proposed 1R/1W STT-MRAM layout (**e**) when width of both transistors is smaller than 6λ, (**f**) when width of either of M1 and M2 is greater than 6λ.

In this section, we present memory cell layouts for the standard single-port STT-MRAM, conventional 1R/1W STT-MRAM, and the proposed 1R/1W STT-MRAM to analyze the bit-cell areas. The cell layout dimensions are evaluated based on λ-based design rules, where λ is half the minimum feature size [17,18]. See the detailed parameters, including minimum metal spacing and minimum metal width, in Figure 8 [19]. In the case of the standard STT-MRAM, the WL runs horizontally across the memory array, and the BL and SL run vertically. If the access transistor width (*W_FET_*) is smaller than 9λ, the horizontal dimension is limited by the metal spacing and the metal width, as seen in Figure 9a [13]:

$$2W_{M2M}+2W_{M}=12\lambda .$$

Otherwise, the horizontal dimension is limited by the transistor width, as seen in Figure 9b:

$$W_{FET}+W_{A2A}=W_{FET}+3\lambda .$$

In the case of the 1R/1W STT-MRAM bit ell, SL is fixed at *G_ND_*, whose metal line can be routed in the horizontal direction, as shown in Figure 9c,d. This can maintain the expression of the x-dimension by having the same number of vertical metal tracks in comparison with the standard STT-MRAM bit cell. However, the two-transistor requirement of 1R/1W STT-MRAM increases the y-dimension by 39%, as follows:

$$2W_{C}+4W_{G2C}+2W_{G}=16\lambda .$$

Figure 9e,f presents the layout of a pair of the proposed 1R/1W dual-port STT-MRAM bit cells. Since the RBL of the odd-column cell and the WBL of the even-column cell are combined into a single bitline (S_BL), the number of vertical metal lines is three, compared to four for the conventional design [11]. This relaxes the minimum horizontal dimension of the cell to 9λ when the width of both M1 (*W_M_*_1_) and M2 (*W_M_*_2_) is smaller than 6λ, as illustrated in Figure 9e. If *W_M_*_1_ > 6λ or *W_M_*_2_ > 6λ, the horizontal dimension, which is determined by the width of the transistor, as shown in Figure 9f, is as follows:

$$max(W_{M1,}W_{M2})+W_{A2A}=max(W_{M1,}W_{M2})+3\lambda .$$

**Figure 10:**
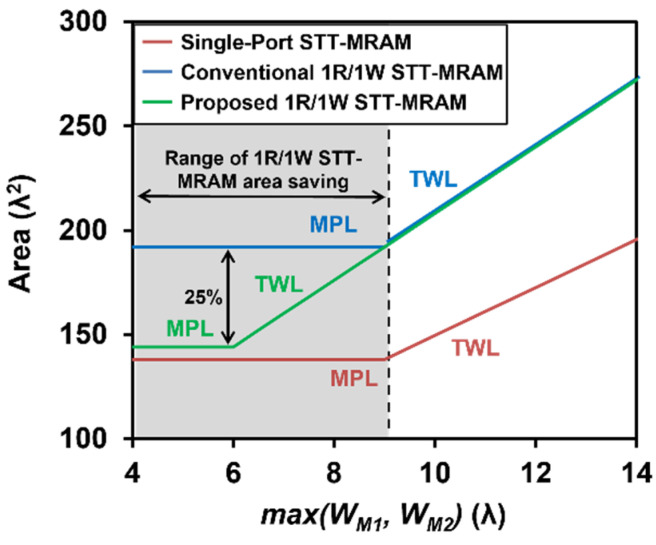
Bit-cell area comparison between single-port STT-MRAM, 1R/1W STT-MRAM, and the proposed MRAM.

Figure 10 shows the bit-cell areas with respect to a range of *max*(*W_M_*_1_, *W_M_*_2_) for single-port STT-MRAM, 1R/1W STT-MRAM, and the proposed 1R/1W design. The bit-cell area is either metal pitch limited (MPL) or transistor width limited (TWL), depending on whether the horizontal dimension is determined by the metal pitch or the transistor width [17]. If *max*(*W_M_*_1_, *W_M_*_1_) < 6λ, the proposed 1R/1W MRAM can improve the bit-cell area by 25% compared with the conventional 1R/1W STT-MRAM. On the other hand, when *max*(*W_RFET_*, *W_WFET_*) is >6λ, the bit-cell area savings diminishes because the proposed design is in the TWL region [13].

## 5. Simulations and Results

To evaluate the proposed memory design in comparison with conventional MRAMs, we utilized a simulation framework [20] that comprises three components:

(1) the Landau–Lifshitz–Gilbert (LLG) equation solver for modeling the magnetization dynamics of a spintronic device [21,22,23];
(2) the non-equilibrium Green’s function (NEGF) formalism in order to determine the resistivity of MTJ [24];
(3) the simulation program with integrated circuit emphasis (SPICE) simulator to model the memory bit-cell circuit.

The LLG equation solver determines the critical current for a 10 ns switching time, based on the parameters in Table 1. The voltage-dependent resistance of the MTJ is obtained by using NEGF formalism [24,25,26]. The resistance function of the spintronic device was coupled with a commercial 45 nm transistor; then, transient SPICE circuit simulations were performed to evaluate the three different memory bit cells.

We designed three different memory bit cells under the identical conditions of 10 ns switching time, 20% write margin (defined as (*I_W_* − *I_C_*)/*I_C_*, *I_W:_* write current, *I_C_*: critical current), and >35% read margin (defined as (*I_R_* − *I_REF_*)/*I_REF_*, *I_R_*: read current, *I_REF_*: reference current) [13]. Specifically, a write voltage and a write access transistor width of each bit cell are determined using the following steps.

(*Step 1*) Set the initial write voltage *V_WRITE_* to 1.0V.

(*Step 2*) Obtain the minimum transistor width *W_M_*_1_ that achieves a write-current driving capability for 10 ns switching time, with a 20% write margin.

(*Step 3*) If *W_M_*_1_ is translated to a metal-pitch limited (MPL) bit-cell area, two sub-steps are subsequently performed:

(*Step 3.1*) Increase *W_M_*_1_ to the maximum width in the MPL region.

(*Step 3.2*) Reduce *V_WRITE_* to the voltage at which the 10 ns switching requirement is met, with a 20% write margin.

The simulation results are presented in Table 2. In the case of conventional single-port and 1R/1W STT-MRAMs, the write access transistor is initially sized at 120 nm (=6λ) after following (*Step 1*) and (*Step 2*). However, the application of (*Step 3*) adjusts the transistor width to 180 nm (=9λ). This is required to improve the dynamic write power consumption by reducing *V_WRITE_* to 0.8V, without any negative impact on the bit-cell area. (See from Figure 10 that the bit-cell area remains the same as the transistor width changes from 6λ to 9λ.) In the case of the proposed design, (*Step 3.1*) and (*Step 3.2*) are not applied because the point of intersection between MPL and TWL is moved to 6λ by the proposed bitline sharing, as shown in Figure 10. This is the reason that the write access transistor width for the proposed bit cell is 120 nm. Accordingly, the proposed design exhibits a 25% smaller area than conventional 1R/1W STT-MRAM.

Note that the proposed design maintains the inherent advantages of the conventional 1R/1W design [10,11]. Because of the dedicated transistor and bitline for the read and write operations, the proposed memory enables us to perform simultaneous read and write accesses. This effectively overcomes the impact of a slow write operation in the overall system performance [10]. Furthermore, the proposed memory can separately optimize the read-access transistor without considering write operations. By using a small access transistor for read operation, it was possible to achieve 15% lower read power consumption and improve the read-disturbance margin (defined as (*I_C_* − *I_R_*/*I_C_*) by ~19%.

## 6. Conclusions

We propose a high-density 1R/1W dual-port STT-MRAM design. Our proposed design combines the RBL of an odd-column cell and the WBL of an even-column cell in the same row, relaxing the minimum achievable area constrained by the metal pitch. The bitline sharing incurs more simultaneous access conflict than the conventional design, owing to the creation of the sneak current or conflicts on the biasing condition of S_BL. However, this can be effectively addressed by using the bit-interleaving architecture with a long interleaving distance. The simulation results reveal that our proposed design improves the memory bit-cell area by 25% compared with that of conventional dual-port design. The proposed 1R/1W MRAM achieves a 15% lower read power and a 19% higher read-disturbance margin than those of the single-port STT-MRAM.

## Figures and Tables

**Table 1:** Parameters of the storage devices.

Device Parameters	Magnetic Tunnel Junction
Activation Energy, *E_A_*	56 k_B_T
Gilbert damping, α	0.01
Saturation magnetization, *M_S_*	1000 × 10^3^ A/m
Dimension of FL (*W_FL_ × L_FL_ × t_FL_*)	40 nm × 40 nm × 2 nm ^1^
Tunneling oxide barrier thickness, *t_MgO_*	1.25 nm
Critical current for 10 ns switching time	27 µA

^1^ The MTJ free layer has an elliptical shape.

**Table 2:** Comparison of three different bit cells in terms of area, power, and read-disturb margin.

	Conventional Single-Port STT-MRAM	Conventional 1R/1W STT-MRAM	Proposed 1R/1W STT-MRAM
Transistor width (nm)	180	180 (M1)/60 (M2)	120 (M1)/60 (M2)
Bit-cell area (µm^2^)	0.0552	0.0768	0.0576
*V_WRITE_* (V)	0.8	0.8	1.0
*V_READ_* (V)	0.2	0.2	0.2
*V_NEG_* (V)	-	−0.8	−0.8
Write Power (µW)	30.15	30.99	32.48
Read Power (µW)	4.00	3.40	3.40
Read-Disturb Margin (%)	48	57	57
